# Treprostinil Supplementation Ameliorates Hepatic Ischemia Reperfusion Injury and Regulates Expression of Hepatic Drug Transporters: An Isolated Perfused Rat Liver (IPRL) Study

**DOI:** 10.1007/s11095-022-03384-x

**Published:** 2022-09-07

**Authors:** Omar Abdulhameed Almazroo, Imam H Shaik, Christopher B Hughes, Abhinav Humar, Raman Venkataramanan

**Affiliations:** 1 Department of Pharmaceutical Sciences, School of Pharmacy, University of Pittsburgh, 3501 Terrace St, Pittsburgh, PA 15219 USA; 2 Thomas Starzl Transplantation Institute, University of Pittsburgh, Pittsburgh, PA USA; 3 Department of Pathology, University of Pittsburgh Medical Center, Pittsburgh, PA USA

**Keywords:** hepatic ischemia reperfusion injury, transporters, treprostinil

## Abstract

**Purpose:**

IR injury is an unavoidable consequence in deceased donor liver transplantation. Cold preservation and warm reperfusion may change the expression and function of drug transporters in the liver due to vasoconstriction, infiltration of neutrophils and release of cytokines. We hypothesize that vasodilation, anti-platelet aggregation and proinflammatory downregulation activities of treprostinil will diminish the IR injury and its associated effects.

**Methods:**

Livers obtained from male SD rats (n = 20) were divided into 1) Control, 2) IR, 3) Treprostinil-1 (preservation only), and 4) Treprostinil-2 (preservation and reperfusion) groups. Control livers were procured and immediately reperfused. Livers in the other groups underwent preservation for 24 h and were reperfused. All the livers were perfused using an Isolated Perfused Rat Liver (IPRL) system. Periodic perfusate, cumulative bile samples and liver tissue at the end of perfusion were collected. Liver injury markers, bile flow rates, m-RNA levels for uptake and efflux transporters (qRT-PCR) were measured.

**Results:**

Cold preservation and warm reperfusion significantly increased the release of AST and ALT in untreated livers. Treprostinil supplementation substantially reduced liver injury. Bile flow rate was significantly improved in treprostinil-2 group. m-RNA levels of Slc10a1, Slc22a1, and Slc22a7 in liver were increased and m-RNA levels of Mdr1a were decreased by IR. Treprostinil treatment increased Abcb11 and Abcg2 m-RNA levels and maintained Slc22a1m-RNA similar to control livers.

**Conclusions:**

Treprostinil treatment significantly reduced liver injury. IR injury changed expression of both uptake and efflux transporters in rat livers. Treprostinil significantly altered the IR injury mediated changes in m-RNA expression of transporters.

## Introduction

Liver transplantation is the only treatment option for several end stage liver diseases. Liver is the second most transplanted solid organ in the US and more than 12,000 patients are on the waiting list for a liver graft. Recent data from national reports show that more than 90% of the transplanted liver allografts were obtained from deceased donors [1, 2]. Hepatic ischemia and reperfusion (IR) injury is an unavoidable consequence in deceased donor liver transplantation, where the liver grafts are subjected to ischemia during organ procurement, preservation, and transportation; and reperfusion injury following transplantation in the recipient. IR injury is associated with vasoconstriction, increased release of pro-inflammatory cytokines and activation of the platelet activating factors (PAF) [3]. IR injury is one of the leading causes of primary graft non-function (PNF) with an incidence of 4–23% after Orthotopic Liver Transplantation (OLT) and may warrant immediate re-transplantation [4]. IR injury is initiated during cold preservation where oxygen and nutrient supplies are low/absent, a period known as cold ischemic phase. The injury is aggravated during warm reperfusion phase with re-oxygenation and availability of nutrients. With advancements in surgical techniques and newer immunosuppressive therapies, the use of livers from marginal donors and extended criteria donors was expanded substantially. Longer preservation times and higher risk factors associated with extended criteria donors make the livers susceptible to profound IR injury and pharmacological approaches focused on reducing the impact of liver IR injury will be helpful in improving the clinical outcomes.

Liver is responsible for clearing a majority of xenobiotics and toxins by converting them to more polar molecules via phase I and II drug metabolizing enzymes and facilitate their elimination [5]. Drug transporters are involved in uptake and efflux of xenobiotics are abundantly expressed in liver. IR injury mediated changes may significantly perturb the function of transporters in the liver.

Oxidative stress associated with chronic kidney disease affects both intestinal and hepatic transporters. Thomson *et al* reported significantly increased systemic exposure of fexofenadine, a nonspecific substrate for hepatic and intestinal drug transporters, in chronic kidney disease patients [6]. Chronic rejection of graft in living donor liver transplant recipients was associated with marked increase in P-gp expression and downregulation of the CYP3A4 expression in the intestines requiring higher doses of cyclosporin for immunosuppression. Additionally, m-RNA expression of pro-inflammatory cytokines related to local inflammation such as COX2, IL-2, IL-6, IL-8, IL-10, and TNF-α were markedly increased [7]. Livers in deceased donor liver transplant (DDLT) have a higher risk of IR injury due to various conditions experienced by the organ during procurement, preservation, transit and surgical manipulation during transplantation.

IR injury causes a substantial reduction the ATP levels in the liver tissue and thereby reduce the activity of energy dependent ATP-binding cassette (ABC) family proteins such as transporters on both apical and basolateral sides [8]. Solute carrier (SLCs) transporters facilitate passage of certain solutes (e.g., sugars, amino acids) across the membrane and actively transport other solutes against their electrochemical gradient by coupling the process with other solutes or ions. The effect of IR injury on ABC and SLC families has been studied using different ischemia reperfusion models and determined to be clinically important [9–12].

Previous studies from our group reported the protective effects of treprostinil treatment to both donors and recipients in rat liver transplantation model and observed vasodilation in liver, higher hepatic blood flow rates, decreased neutrophil infiltration, decreased expression of adhesion molecules, and lower levels of circulating proinflammatory cytokines [13]. The protective effects were observed when treprostinil was given to rat OLT recipients only, but the magnitude of effect was lower. It is not practical to treat an organ donor before procurement due to multiple clinical and practical reasons. However, addition of treprostinil to the organ preservation solution (UW) may ameliorate the IR injury and influence the expression and activity of primary drug transporters in liver.

We hypothesized that vasodilation, anti-platelet aggregation and proinflammatory downregulation activities of treprostinil will diminish the IR injury associated effects. The goal of our study was to characterize the differential expression of major hepatic uptake (Slc22a7/Oat2, Slc22a1/Oct1, Slc10a1/Ntcp, Slco1a1/Oatp1a1, Slco1a4/Oatp1a4 and Slco1b2/Oatp1b2) and efflux (Abcb1/Mdr1a, Abcc2/Mrp2, Abcb11/Bsep and Abcg2/Bcrp) drug transporters after IR injury and document the protective effects of supplementing treprostinil in the UW solution and during reperfusion using Isolated Perfused Rat Liver (IPRL) system.

## Materials and Methods

### Chemicals

CoStorSol™ (University of Wisconsin; UW) cold organ preservation solution purchased from Preservation Solutions Inc. (Elkhorn, WI). QIA shredder and RNeasy Mini kits were purchased from QIAGEN (Hilden, Germany). iScript™ Reverse Transcription Super mix for RT-qPCR was purchased from Bio-Rad Laboratories, Inc. (Hercules, CA). TaqMan primers for drug transporters and housekeeping genes were purchased from Life Technologies (Carlsbad, CA). E-Gel^(R)^ EX agarose gel (4%), 10 bp DNA Ladder, E-Gel® iBase™ and E-Gel® Safe Imager™ Combo Kit were purchased from Thermo Fisher Scientific Inc. (Waltham, MA). Aspartate aminotransferase (AST) and alanine aminotransferase (ALT) assay kits were from Pointe Scientific, Inc. (Canton, MI). Treprostinil (Remodulin^(R)^; 1 mg per mL) was provided by United Therapeutics Corporation (Silver Spring, MD). Isolated Perfused Rat Liver system was obtained from Radnoti LLC (Covina, CA). All chemicals and reagents were of highest purity and obtained from commercial sources.

### Animals

Adult male Sprague Dawley (SD) rats (225–250 g) were procured from Charles River Laboratories, Inc. (Wilmington, Massachusetts). Rats were housed in animal facility maintained with a 12 hours’ light and dark cycle and had free access to food and water. All the study procedures were in accordance with the protocol approved by the University of Pittsburgh Institutional Animal Care and Use Committee and were consistent with the Guide for the Care and Use of Laboratory Animals (National Research Council, 2011, Washington, District of Columbia).

### Study Design

A total of 20 rats were randomly assigned to the following study groups: 1) Naïve (n = 3): livers were procured and frozen immediately for m-RNA analysis; 2) Control (n = 4): livers were procured and perfused immediately; 3) Ischemia-Reperfusion group (n = 5): livers were harvested, cold preserved (4°C) in UW solution for 24 hours followed by reperfusion; 4) Treprostinil-1 (n = 4): rat livers were harvested and preserved in UW solution supplemented with treprostinil (20 ng/mL) for 24 hours and flushed with (~20 mL) ringers lactate solution with 20 ng/mL treprostinil and reperfused without treprostinil; 4) Treprostinil-2 (n = 4): The livers were subjected to procedures similar to the group 4 and additionally treprostinil (20 ng/mL) was added during perfusion. All the livers were perfused using IPRL system for 2 hours in the recirculation mode.

### Surgical Procedure

The procedures for procuring, preserving, and perfusing the livers have been described previously [14–16]. Briefly, rats were anesthetized using isoflurane and the depth of anesthesia was checked by toe pinch reflex. The abdominal cavity of the rat was opened with a V-shaped incision to expose the visceral organs. The bile duct was cannulated with PE10 tubing and the portal vein and external hepatic vein were cannulated with catheters for inlet and outlet of the perfusate, respectively. For groups requiring preservation, livers were perfused with 30 mL UW solution (with or without treprostinil) and harvested. The isolated rat livers were preserved in UW solution (with or without treprostinil) for 24 hours at 4°C. Livers were flushed with 30 mL cold ringers lactate solution and mounted on to IPRL system and equilibrated for 10 min and the perfusion was converted to recirculation mode. The perfusion was carried out for 120 minutes with Krebs–Henseleit bicarbonate buffer (saturated with 95%/5% O_2_/CO_2_ and supplemented with sodium taurocholate (4.75 mg/L) as continuous infusion) at 3 mL/min/g liver. Periodic outlet perfusate samples and cumulative bile samples were collected in pre-weighted centrifuge tubes at 30-minute intervals. At the end of perfusion, a piece of the liver was preserved in formalin for histological evaluation and rest of the liver tissue was flash frozen immediately in liquid nitrogen and stored at -80°C until further analysis.

### Determination of Liver Injury Biomarkers

Aspartate aminotransferase and Alanine aminotransferase levels in the perfusate were measured using commercially available kits.

### Other Hepatic Graft Assessments

Volume of bile collected was assessed by gravimetry and bile flow rates were calculated. Wet liver weight was recorded at the end of the perfusion and the was expressed as a percentage of the total body weight (% TBW).

### Tissue Histology Staining

The formalin fixed liver tissues were processed for H&E staining and the histological sections were evaluated by pathologist blinded to the study groups. The necrotic areas were evaluated using morphometric analysis estimation from 3 randomly selected areas per H&E section.

### mRNA Isolation and Purification

The mRNA extraction was performed as per the kit instructions. Briefly, ~30 mg of frozen liver tissue was transferred to a centrifuge tube and 600 μL RNeasy RLT buffer was added and immediately homogenized using a conventional rotor–stator homogenizer. Tissue homogenate was transferred to QIA shredder column placed in a 2 mL tube and centrifuged at maximum speed for 2 minutes then another 3 minutes after removing the column. Supernatant was transferred to a centrifuge tube and mixed with 600 μL 70% ethanol. The mixture was loaded on to RNeasy spin column in 2 steps (700 μL then 500 μL) and centrifuged for 15 seconds at 12,000 rpm. RNeasy column was cleaned with 700 μL with RW1 buffer (15 seconds at 12,000 rpm) and then two times with 500 μL RPE buffer (15 seconds at 12,000 rpm). Total mRNA was eluted into 80 μL RNase-free distilled water by centrifugation (1 minute at 12,000 rpm) and stored at -80°C for further analysis.

### Quantification and Assessment mRNA Purity and Integrity

mRNA yield and purity were assessed using Nano Drop Spectrophotometer. The absorbance difference at 260/280 nm provides both concentration and purity of the sample. Agilent RNA 6000 nano kit and Agilent 2100 Bioanalyzer were used to evaluate the integrity of the mRNA in samples. The samples were processed as per the kit instructions. The bioanalyzer reading provides RNA Integrity Number (RIN) for each sample [17, 18].

### cDNA Preparation and Real Time qPCR

iScript™ Reverse Transcription Super mix for RT-qPCR was used to prepare cDNA. Briefly, 1 μg of total RNA, 4 μL iScript RT super mix and RNase free water was added to bring the volume to 20 μL. The mixture was incubated in the following thermal cycles; 5 minutes at 25°C for priming; 30 minutes at 42°C to reverse transcriptase; then 5 minutes at 85°C to inactivate the transcriptase enzyme. The final cDNA yield was diluted with 80 μL RNase free water. Real-time PCR reaction was performed in 96 well plate that contained 20 μL of the reaction mix per well. Each reaction mixture contained 1 μL TaqMan primer, 10 μL TaqMan master mix, 4 μL cDNA and 5 μL RNase-free water. Plates were covered and centrifuged at 3000 rpm (4°C) for 3 minutes. Applied Biosystems® 7500 Real-Time PCR System was used to amplify and detect targeted genes. Initially, uracil DNA glycosylase (UDG) incubation was performed at 50°C for 2 minutes, then AmpliTaq Gold® enzyme activation at 95°C for 10 minutes, followed by 40 cycles of 95°C for 15 seconds amplifications and 60°C for 1 minute to extend the DNA strands. All genes were analyzed in the same plate for each sample to minimize inter run variability.

### Primer Efficiency and Specificity

Serial dilutions of the pooled cDNA samples described above were tested to check the efficiency of each primer. We evaluated four-fold dilution for primers with cycle threshold (Ct) around 24- and two-fold dilution for primers with Ct around 30. Each dilution level was run in triplicate and all dilutions for each primer were run in the same plate to minimize inter-run variability.

Samples from the amplified RT-qPCR wells were separated on 4% agarose gel to identify and confirm the primer identities by size. Samples and ladder were loaded into the wells of pre-casted E-Gel EX 4% agarose gel, placed on iBase™ and separated by electrophoresis. Bands were visualized and photographed by the E-Gel® Safe Imager™.

### Data Analysis

The area under the perfusate concentration-time curve (AUC) for ALT and AST was calculated using linear trapezoidal method. The differences between the groups were compared using Analysis of Variance (ANOVA), followed by Dunnett’s post hoc test using control group as reference. Standard curve for each primer was generated to assess the efficiency. The amplification efficiency (E) is calculated from the slope of the standard curve using the equation; E = 10^–1/slope^_._ Amplification efficiency (E) was converted to percentage efficiency (%E) using the equation; %E = (E – 1) × 100. The relative levels of transporter mRNA was normalized to copy numbers of β–actin mRNA. The relative fold change of mRNA for all genes was quantified using the 2^-ΔΔCt^ method [19]. Groups were compared using Analysis of Variance (ANOVA) followed by Bonferroni post-hoc analysis. p ≤ 0.05 were considered significant. All the data is presented as Mean ± SE.

## Results

The wet weight for livers at end of perfusion in all the groups was below 4% total body weight and the samples from all the livers were used for further analysis. The portal vein back pressure was monitored during the liver perfusion in all groups and the portal vein pressure did not change during the perfusion.

### Histology

Histological evaluation of H&E-stained liver sections from control livers showed no alterations to the liver morphology. Livers in IR group which underwent 24 hours of preservation and IPRL perfusion for 2 hours showed pallor at zone 3 with minimal damage to hepatocytes and addition of treprostinil did not alter these morphological changes in the livers.

#### Biliary Flow Rates and Hepatic Microcirculatory Integrity

**Fig. 1:**
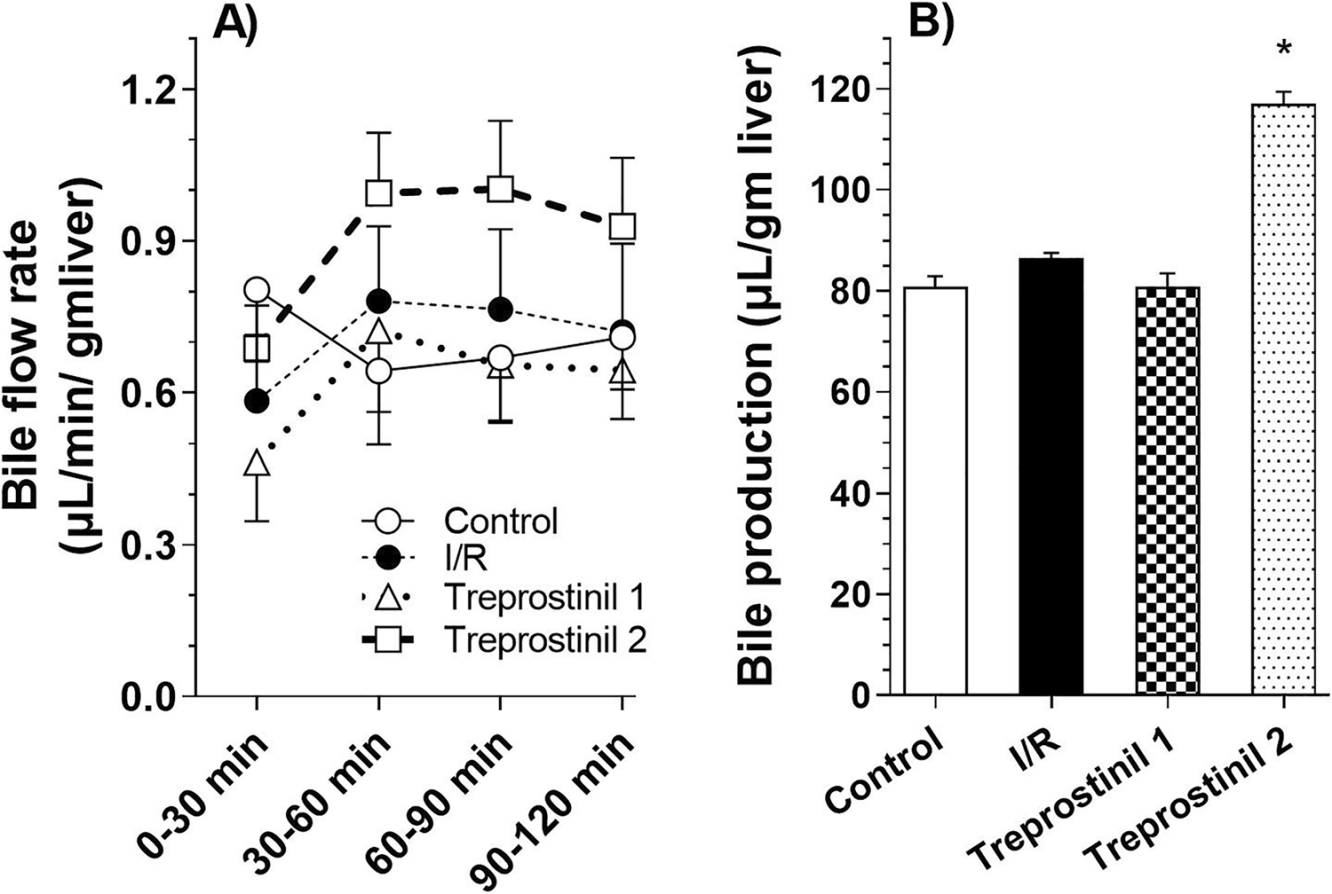
Bile flow rate and total bile produced normalized to liver weight during 2-hour perfusion using IPRL. (**A**) Bile flow rates normalized to liver weight. (**B**) Bile production normalized to liver weight. All the perfusions were performed for 2 hours with KH buffer saturated with 95%/5% O_2_/CO_2_ and supplemented with sodium taurocholate (4.75 mg/L). Control (livers were harvested and perfused; no preservation, no treatment during IPRL), ischemia/reperfusion group (24 hour cold preservation in UW solution; no treatment during preservation and IPRL perfusion), Treprostinil 1 (livers were preserved for 24 hours in UW solution supplemented with 20 ng/mL treprostinil and perfused; no treatment during IPRL perfusion) and Treprostinil 2 (livers were preserved for 24 hours in UW solution supplemented with 20 ng/mL treprostinil and perfused with buffer supplemented with 20 ng/mL treprostinil). Data are presented as Mean ± SE (n = 4–5/group). *p ≤ 0.05 compared to control group.

The cumulative bile samples collected over 30-minute intervals were measured by gravimetry. Bile flow rate for each interval (panel A) and total bile collected (panel B) were calculated for each group are presented in Fig. 1. The bile flow rate and total amount of bile produced from control livers was consistent with literature values reported for IPRL studies using healthy livers indicating the utility of the model for further studies. Bile flow rate from livers in IR group and those perfused with addition of treprostinil (20 ng/mL) were similar to control group. However, the addition of treprostinil (20 ng/mL) during both preservation and reperfusion significantly increased the total bile collected by 44.7% as compared to control livers. The average difference in portal vein backpressure during IPRL reperfusion was similar in all the study groups.

#### Hepatic Injury Biomarkers

**Fig. 2:**
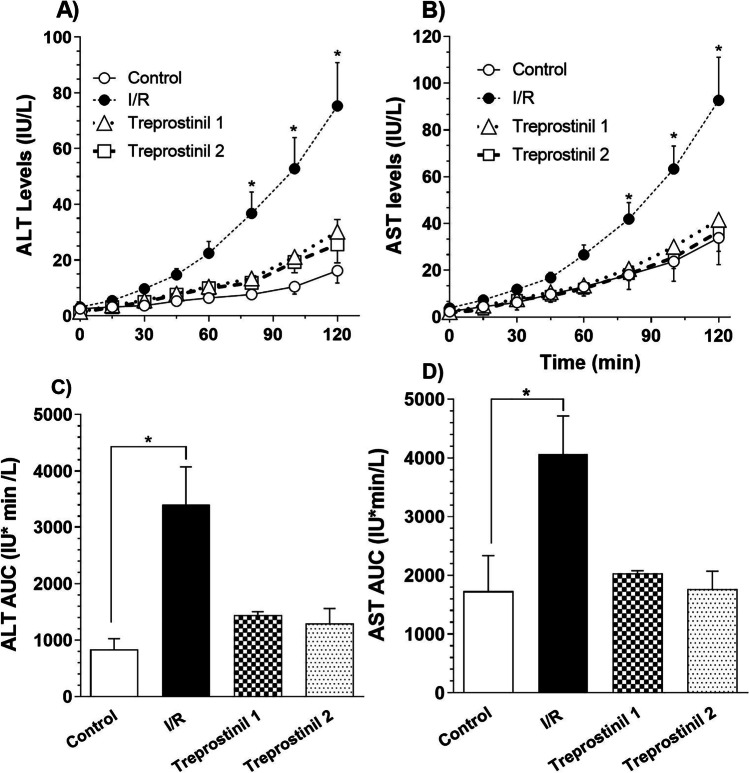
Time course of perfusate ALT and AST levels and Area Under the Curve for ALT and AST *vs* time. ALT (**A**) and AST (**B**) levels over 2 hours IPRL reperfusion. Area under the perfusate ALT (**C**) and AST (**D**) concentration-time curve (AUC; IU/L/min). Control (no preservation, no treatment during IPRL), ischemia/reperfusion group (Preservation, no treatment during preservation and IPRL perfusion), Treprostinil 1 (20 ng/mL treprostinil during preservation and no treatment during preservation and IPRL perfusion) and Treprostinil 2 (20 ng/mL treprostinil during preservation and IPRL perfusion). Results are expressed as mean ± SEM (n = 4–5/group). * p ≤ 0.05 compared to control group.

Levels of liver injury markers, ALT and AST, were analyzed in periodic perfusate samples are presented in Fig. 2. Panel A shows the time course of the ALT levels in perfusate. The ALT release from control livers (open circles) was low for the duration of perfusion indicating minimal injury due to the liver. The levels in IR group (filled circles) were initially low and increased gradually with time. The perfusate ALT levels in IR group at 80 minutes and onwards were significantly higher (P < 0.05) than control group at corresponding time points. Treprostinil addition to the UW solution during preservation (treprostinil-1; open triangle) decreased the IR induced liver injury and the ALT levels in the perfusate were maintained similar to the control livers. Additionally, supplementation of treprostinil in the preservation solution and in perfusate (Treprostinil-2 group; open square) showed the ALT levels similar to the control group. Panel B shows the time course of AST release in perfusate in the study groups. AST levels in the control livers were low for the duration of IPRL perfusion and IR group showed a significant increase (P < 0.05) in AST release in the perfusate from 80 min onwards as compared to the control group. Addition of treprostinil during preservation or preservation and perfusion substantially decreased the release of hepatic injury markers and the profiles were similar to control livers.

The area under the perfusate levels *vs* time curve for ALT and AST are presented in panels C and D, respectively. The AUC for ALT in the control group was 835 IU/L/min and the IR resulted in a statistically significant (4-fold) increase in AUC. Addition on treprostinil during preservation or preservation and reperfusion substantially reduced the ALT levels in perfusate and the AUC values were maintained similar to the control group. Similarly, the area under the curve for perfusate AST levels *vs* time was significantly increased by 2.4 times (P < 0.05), in the IR injury group, when compared to control group. Treprostinil supplementation during preservation or preservation and reperfusion substantially reduced AST levels in perfusate and the AUC values were similar to the control livers.

### mRNA Isolation and Quality Assessment

mRNA isolated from liver samples were assessed by several methods to assure that the real-time quantitative polymerase chain reactions (RT-qPCR) are appropriate and reproducible. Nano drop spectrophotometer was used to quantify mRNA concentrations and to assess sample purity. mRNA purity was assessed as the absorbance ratio at 260/280 nm and 260/230 nm. Ratios of 1.8 to 2.2 are generally considered to indicate purity and acceptable yield and the ratios for our samples were 2.05 ± 0.02 and 2.08 ± 0.17 for 260/280 nm and 260/230 nm, respectively indicating high purity. The integrity of mRNA was assessed using electrophoresis, where 18S and 28S fractions are separated and quantified by Agilent 2100 Bioanalyzer and an algorithm was used to calculate the RNA Integrity Number (RIN) from the information gathered from sample electropherograms. The RIN values for all the samples were observed between 7 and 9.6, indicating good integrity for isolated m-RNA.

### Primers Efficiency

Validated TaqMan primers were obtained for ten hepatic drug transporters and two housekeeping genes. List of the names, symbols, amplicon sizes and efficiencies of the gene primers are presented in Table I. Post amplification, all genes were verified by electrophoresis of RT-qPCR products on 4% agarose gels to ensure specificity and size of each amplicon. Additionally, gels were visually examined for any extra bands. Standard curves generated by plotting cycle threshold (Ct) *versus* the log value of the serial concentrations were used to quantify the primer efficiency. Standard curves for all the primers were linear (R^2^ > 0.99) and the slopes were used to calculate the efficiency using the equations described earlier in the methods section. The percentage efficiency values for the primers used were between 90 and 100%, except for Bcrp (Abcg2; 84.3%) and Gapdh (84.7%). The obtained data met or exceed the guidelines set for minimum information needed for publication of quantitative real-time PCR experiments (MIQE).

**Table I:** List of Genes, Symbol, Amplicon Size and Amplification Efficiency of Primers used. Efficiency is Calculated by Serial Dilutions. All Other Information is Supplied with TaqMan Primers

Gene	Gene symbol	Assay ID	Amplicon size	Efficiency
Glyceraldehyde-3-Phosphate Dehydrogenase	Gapdh	Rn01775763_g1	174	84.7%
β-actin	LOC681152	Rn01424440_s1	93	92.7%
Multi-drug resistance 1a (Mdr1a)	Abcba1	Rn01639253_m1	79	93.8%
Multidrug resistance-associated protein 2 (Mrp2)	Abcc2	Rn00563231_m1	60	95.5%
Breast cancer resistance protein (Bcrp)	Abcg2	Rn00710585_m1	94	84.3%
Bile salt export pump (Bsep)	Abcb11	Rn01515444_m1	71	90.8%
Na^+^-taurocholate co-transporting polypeptide (Ntcp)	Slc10a1	Rn00566894_m1	63	90.6%
Organic cation transporter 1 (Oct1)	Slc22a1	Rn00562250_m1	54	92.5%
Organic anion transporter 2 (Oat2)	Slc22a7	Rn00585513_m1	54	95.9%
Organic anion-transporting polypeptide 1a1 (Oatp1a1)	Slco1a1	Rn01463125_m1	93	99.8%
Organic anion-transporting polypeptide 1a4 (Oatp1a4)	Slco1a4	Rn00756233_m1	134	94.5%
Organic anion-transporting polypeptide 1b2 (Oatp1b2)	Slco1b2	Rn01492635_m1	79	90.1%

### Expression of Uptake Transporters

**Fig. 3:**
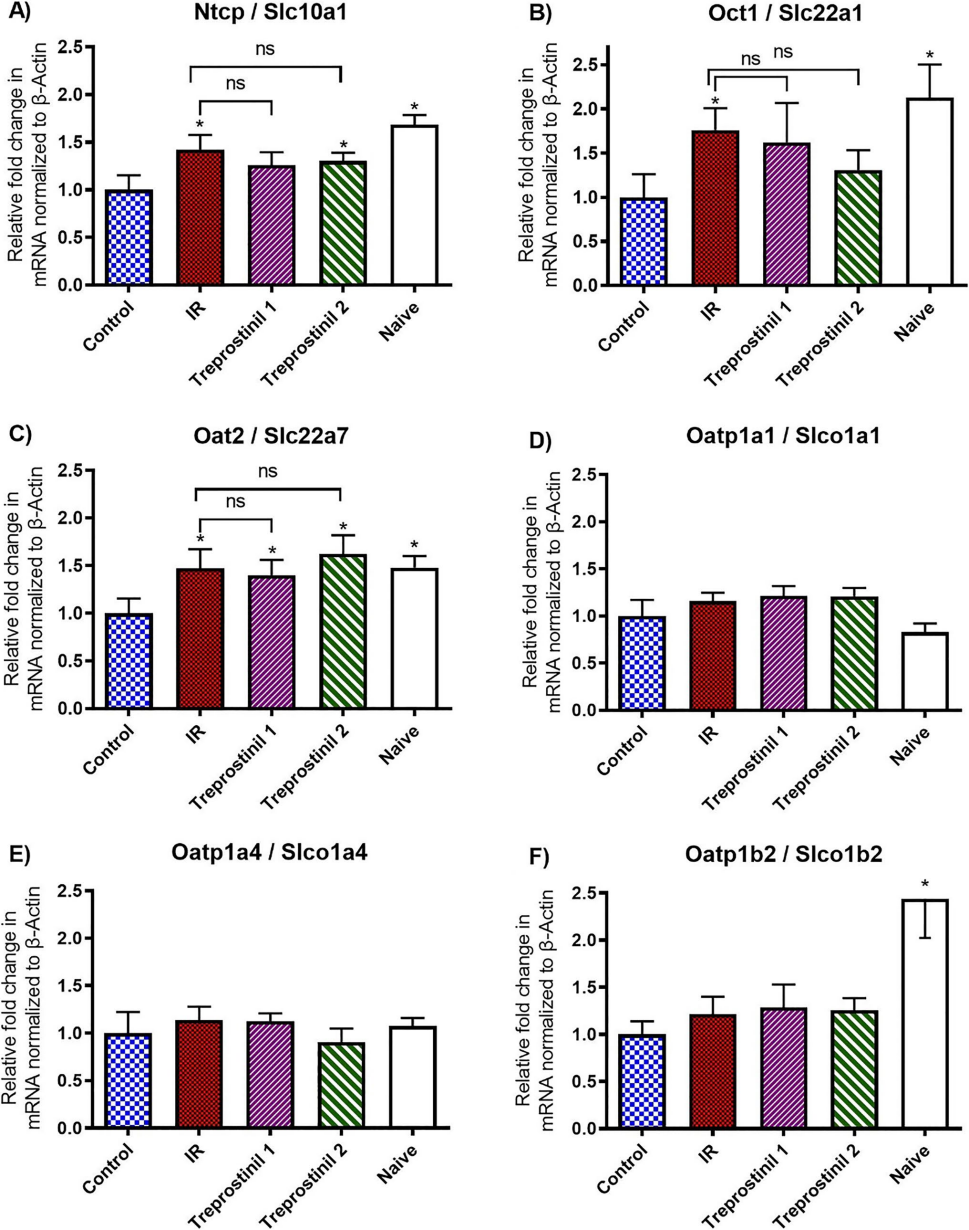
Relative expression of uptake transporters in liver tissue collected post perfusion. Expression of Slc10a1, Slc22a1, Slc22a7, Slco1a1, Slco1a4 and Slco1b2 mRNA were measured by RT-qPCR and normalized to the mRNA expression of β-actin in corresponding sample. Each sample was analyzed in triplicate (n = 4/ group). Results are expressed as mean ± SEM. * p ≤ 0.05 compared to control; NS = Statistically not significant for respective comparisons.

The effect of twenty-four hours cold preservation and two hours of warm IPRL on uptake drug transporters gene expression were measured using RT-qPCR and the data is presented in Fig. 3. Livers from naïve rats were also analyzed with the study groups. Control livers showed a significant downregulation of Slc10a1/Ntcp, Slc22a1/Oct1, Slc22a7/Oat2 and Slco1b2/Oatp1b2 expression by at least ~40%, when compared to naïve livers indicating the impact of ex-vivo perfusion on these transporters. The reduced Slco1b2/Oatp1b2 expression due to IPRL procedure was not affected due to preservation and reperfusion with or without treprostinil. However, expression of Slco1a1/Oatp1a1 and Slco1a4/Oatp1a4 was not affected by either IPRL or study treatments.

The livers in the IR group showed significant increase in the mRNA expression of Slc10a1/Ntcp, Slc22a1/Oct1 and Slc22a7/Oat2 by 1.42, 1.76 and 1.47 folds respectively, compared to the control group. Addition of treprostinil (20 ng/mL) to the UW solution during preservation and /or reperfusion reduced the IR mediated effects on expression of Slc10a1/Ntcp and Slc22a1/Oct1. The mRNA expression of Slc10a1/Ntcp was not significantly different between the IR group and treprostinil treated groups indicating that the IR mediated upregulation of Slc22a7/Oat2 expression was not affected by treprostinil.

### Expression of Liver Canalicular Membrane Transporters

**Fig. 4:**
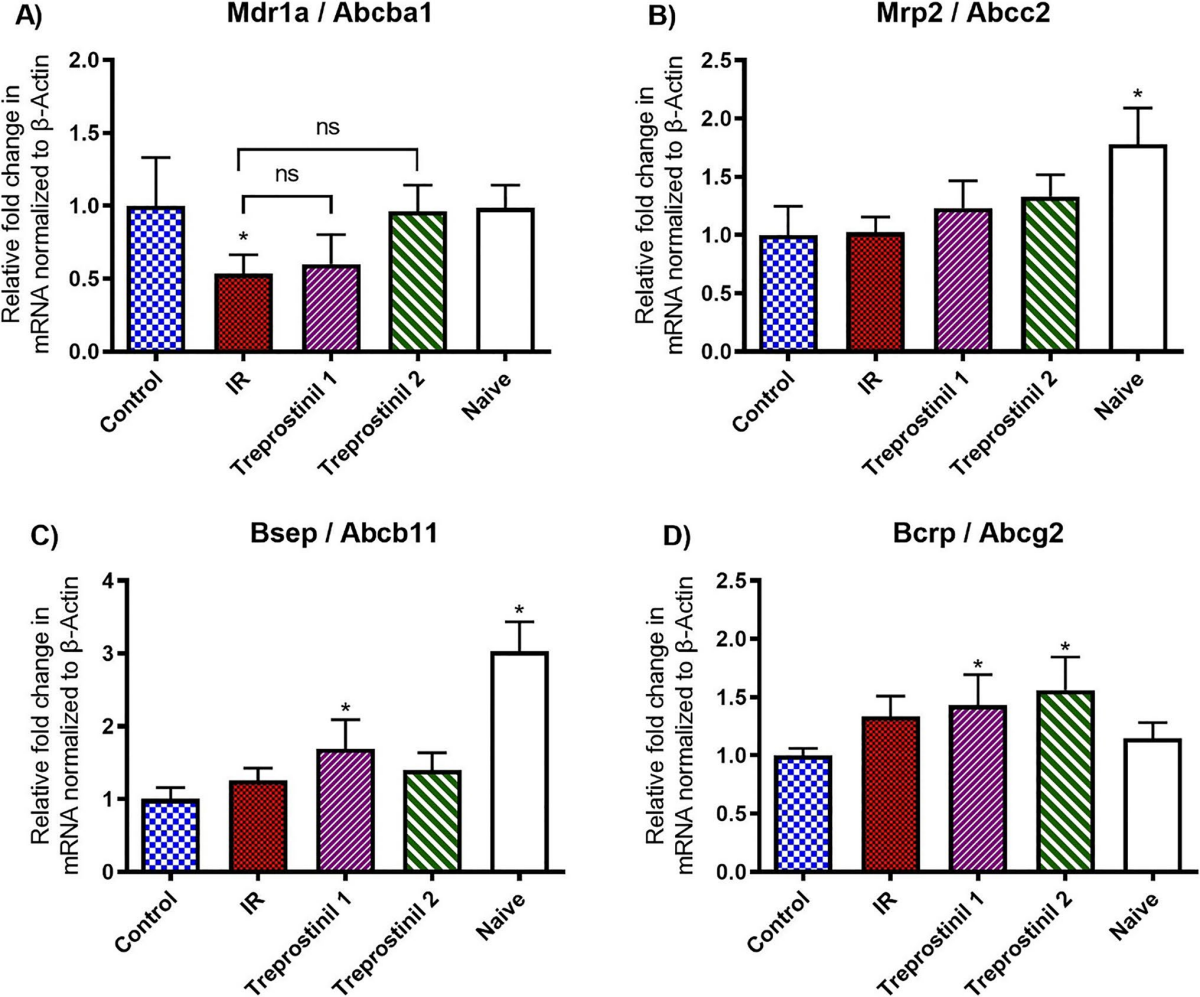
Relative expression of efflux transporters in liver tissue collected post perfusion. Expression of Abcb1, Abcc2, Abcb11 and Abcg2 mRNA were measured by RT-qPCR and normalized to the mRNA expression of β-actin in corresponding sample. Each sample was analyzed in triplicate (n = 4/ group). Results are expressed as mean ± SEM. *p ≤ 0.05 compared to control; NS = Statistically not significant for respective comparisons.

The expression of canalicular membrane transporters in naïve livers and livers in different study groups is presented in Fig. 4. Expression of Mdr1a (P-gp; *Abcb1*) and Bcrp (Abcg2) in livers from naïve and control groups were similar indicating that IPRL procedure did not affect their expression. However, Mrp2 and Bsep expression was significantly reduced by 67% and 44% respectively in control livers as compared to Naïve group.

Multidrug resistance protein 1a (Mdr1a; P-gp; *Abcb1*) expression was significantly reduced (46.7%) in IR group as compared to control livers and addition of treprostinil attenuated the effect of IR. Mdr1a mRNA levels were maintained similar to control group with treprostinil supplementation during preservation as well as preservation and reperfusion. We did not observe a statistically significant difference when Mdr1a mRNA levels in IR group were compared individually to treprostinil treated groups indicating that the IR mediated downregulation of Mdr1a expression was not affected by treprostinil.

 Treprostinil addition to preservation solution showed a small but significant increase in m-RNA for Bsep as compared to control group. However, treprostinil supplementation during preservation and reperfusion increased Bsep expression, but the increase was not statistically significant. The expression of breast cancer resistance protein (Bcrp; Abcg2) mRNA was not altered after IPRL (control) when compared to naïve livers. Though not significant, Bcrp levels were increased by 25% in IR group as compared to control group. Furthermore, addition of treprostinil (20 ng/mL) during preservation or preservation and reperfusion showed a significant increase in the expression of Abcg2 (Bcrp) by 45% and 56% respectively.

## Discussion

The hepato-protective effects of treprostinil supplementation during preservation and reperfusion was studied. Additionally, effects of treprostinil addition on the expression of hepatic transporters is also presented. IR injury is an unavoidable consequence encountered during liver transplantation which manifests as an increase in pro-inflammatory cytokine levels and release of excessive amounts of reactive oxygen species leading to increased morbidity and organ dysfunction [20–22]. IR injury to liver may also be precipitated by trauma during surgical resection or transplantation and by infection. Though many approaches have been studied to counter the deleterious effects of hepatic IR injury, currently there is no pharmacological intervention available to treat the IR injury. The observations from current study and previous reports from our group indicate that treprostinil shows protection against IR injury to the liver [13].

Liver injury after IR results in release of intracellular amino transferases and significantly lowers the bile flow [15, 16, 23]. Pretreating both donors and recipients of rat liver transplantation with treprostinil reduced the circulatory levels of transaminases and TNF-α; and hepatic blood flow was substantially increased [13]. IR injury to the liver is also known to alter phase I, and II drug metabolizing enzymes and transporters in both apical and basolateral membranes in the hepatocytes. However, the magnitude of injury varies substantially with animal model, duration of ischemia (short *versus* long) and/or reperfusion, and enzymatic pathways being studied [24–26].

Isolated rat liver perfusion (IPRL) is a well-established ex-vivo model to study physiological states and pathological conditions such as IR injury [27, 28]. IPRL gives the flexibility to collect periodic perfusate and bile samples, simultaneously. Livers were cold preserved in UW solution with and without treprostinil for 24 h, perfused with Krebs-Henseleit bicarbonate buffer in recirculation mode for 2 h. Perfusate and bile samples were utilized for estimating injury biomarkers, and canalicular membrane integrity and secretory functions respectively. The selected model mimics conditions experienced by the liver during transplantation and the observed increase in the levels of AST and ALT are comparable to literature reports with an increase in the AUCs of ALT and AST by 4.5 and 3 folds, respectively [23].

The intensity of IR injury is substantially affected by time of pharmacological intervention and reports show that prostaglandins treatment of the donors before harvesting the liver has shown favorable outcomes after transplantation [29, 30]. L-Arginine (400 mg/kg) treatment of liver during 6 h cold preservation showed significant reduction (12.5%) in biliary tract complications after OLT in pigs [31]. Previous studies in rat OLT model observed reduced levels of transaminases, TNF-α, INF- γ, and an increase in the hepatic blood flow to the livers when both donors and recipients were treated with treprostinil [13]. In the current study, supplementing treprostinil in the UW solution during cold preservation significantly decreases the release of intracellular liver enzymes. Furthermore, this is the first study to report promising outcomes with addition of a pharmacological agent to the UW solution during cold preservation. Our approach is suitable and practical in the clinic, where a donor organ can be transported in the preservation solution supplemented with treprostinil and beneficial effects can be observed post transplantation.

Cholangitis, inflammation of the biliary tract, is one of the most common complications observed after OLT which results in reduced bile flow. Parasrampuria *et al* reported a 40% reduction in bile flow in livers with arterial deprivation and showed that number of bile duct sections were identical between naïve and IR groups suggesting that drug transporters expression and/or activity may be affected due to reduced blood flow to the liver [32]. Ikeda *et al* reported a significant reduction in post-transplant bile flow with increase in both cold ischemia and warm ischemia times [23]. Using IPRL studies, Chimalakonda *et al* reported that livers pretreated with methylprednisolone decreased the release of TNF-α but the bile formation was also decreased [15]. Thorling *et al* conducted longitudinal studies in rats using *in vivo* partial warm ischemia model and show reduced bile flow immediately after reperfusion. The recovery of bile flow was observed after 48 h of reperfusion [26]. The addition of treprostinil (20 ng/mL) during preservation and reperfusion increased the bile flow suggesting its role in reducing cholangitis. Our findings show that treprostinil treatment significantly increases bile production after cold ischemia and warm reperfusion of the liver.

The expression of apical and basolateral drug transporters in the liver tissue was assessed to elucidate our model and the identify effect of treprostinil treatment on the functional capacity of the liver. Literature reports show that both uptake and efflux transporters are affected to different degrees due to ischemia using various *in vitro* and *in vivo* models. Fouassier *et al* reported a 2 – 5fold reduction in the expression of Slc10a1/Ntcp, Abcb11/Bsep, and Abcc2/Mrp2 mRNA in livers at 24 hours after arterial ischemia as well as hepatocyte hypoxia model. Importantly, Cystic Fibrosis Transmembrane Conductance Regulator (Cftr) mRNA, a major transporter in cholangiocytes was increased by four folds in the ischemic livers. Hypoxic hepatocytes showed a 60% reduction in mRNA expression of nuclear factors; hepatic nuclear factor (HNF4α), retinoid X receptor (RXRα), and farnesoid X receptor (FXR) [32]. Wagner *et al* reported the use of inducers such as phenobarbital and rifampicin for activation of nuclear receptors to treat cholestasis in humans [33]. Tanaka *et al* showed significantly increased cytokine levels and reduced expression of both uptake and efflux transporters using a partial liver ischemia and reperfusion model. Interestingly, Mdr1b mRNA was increased but HNF4α protein levels were reduced. Additionally, depletion of Kupffer cells with GdCl_3_ abolished these IR induced effects [34]. Duration of cold preservation also effects on expression of drug transporters mRNA and proteins substantially after OLT in rats. The mRNA levels of ILBP, Ostα, Ostβ, and FXR were dramatically decreased in livers undergoing 12 h *vs* 1 h cold preservation at 1, 3 and 7 days after transplantation indicating the effect of both ischemia and reperfusion durations on the magnitude of IR injury to liver [35]. Inflammation associated with infection and sepsis resulted in a significant downregulation of hepatic drug transporters in humans as well as rats [36, 37].

Our studies show that prolonged cold ischemia and acute warm reperfusion induced gene expression for solute carrier transporters Slc10a1/Ntcp, Slc22a1/Oct1 and Scl22a7/Oat2. However, Mdr1a/Abcba1 (P-gp) expression was significantly decreased. This phenomenon is in line with the observed reduction in expression and activity of the P-gp in livers subjected to partial ischemia, and exposed to endotoxins (lipopolysaccharide (LPS)) or IL-6 [8, 38, 39]. Thorling *et al* observed a significant downregulation of P-gp (Mdr1a; Abcb1) after prolonged IR injury [26]. Zhu *et al* studied bile duct injury in rat autologous liver transplantation and identified that warm ischemia time longer than 20 min causes significant injury to the biliary ducts with an associated increase in biliary epithelial cell apoptosis index [40]. Altered expression of drug transporter genes and proteins and associated changes in the activity of drug transporters could substantially affect the therapeutic outcomes of many essential medications are substrates of both p450 enzymes as well as drug transporters.

In our studies addition of treprostinil, a synthetic stable prostacyclin (PGI_2_), prevented the upregulation of the uptake transporters (both sodium taurocholate co-transporting polypeptide and organic cation transporter 1). Treprostinil treatment during preservation and reperfusion showed sustained reduction of Oct1 expression. Expression of apical efflux transporter, P-gp (Mdr1a; Abcb1a) was significantly reduced by IR and addition of treprostinil reversed the affect and the levels were similar to control group. Our findings are in line with observations from rat liver transplantation studies reported by Ghonem *et al* [13] indicating the validity of *in situ* perfusion model for hepatic IR injury studies.

Protective effects of prostaglandins and prostacyclins have been studied previously in both preclinical and clinical settings [41–44]. It is well-documented that PGI_2_ elicits protective effects through anti-platelet aggregation and inhibition of leukocyte adhesion to endothelial cells [45]. The current study reports protective effects of treprostinil addition to preservation solution through reduction in release of intracellular enzymes during reperfusion, increased bile flow and modulation of hepatic drug transporter expression. Though these findings indicate improved function of liver, further studies are needed to elucidate the effect of treprostinil addition on the activity and expression of both drug metabolizing enzymes and drug transporters.

In conclusion, the current study demonstrates the effect of cold ischemia and warm perfusion on the expression of basolateral (SLC; uptake) and apical (ABC; efflux) hepatic drug transporters using an isolated rat liver perfusion system. The transcription of important drug transporter genes was significantly affected. However, addition of treprostinil normalized the changes and improved function of liver grafts. Though the presence of treprostinil during preservation showed positive outcomes, addition of treprostinil during both preservation and reperfusion seems to show better outcomes and possibly improve the use of organs from marginal and extended criteria donors. Further studies are needed to study the effects of treprostinil addition on the function of drug metabolizing enzymes and drug transporters.
